# Sf-FGFR and Sf-SR-C Are Not the Receptors for Vip3Aa to Exert Insecticidal Toxicity in *Spodoptera frugiperda*

**DOI:** 10.3390/insects13060547

**Published:** 2022-06-14

**Authors:** Yinxue Shan, Minghui Jin, Swapan Chakrabarty, Bo Yang, Qi Li, Ying Cheng, Lei Zhang, Yutao Xiao

**Affiliations:** Shenzhen Branch, Guangdong Laboratory of Lingnan Modern Agriculture, Genome Analysis Laboratory of the Ministry of Agriculture and Rural Affairs, Agricultural Genomics Institute at Shenzhen, Chinese Academy of Agricultural Sciences, Shenzhen 518120, China; shanyinxue9@163.com (Y.S.); jinminghui@caas.cn (M.J.); swapan.ag.sau@gmail.com (S.C.); yangb19940629@163.com (B.Y.); liqi9150@163.com (Q.L.); canoecheng@163.com (Y.C.)

**Keywords:** *Spodoptera frugiperda*, *Bacillus thuringiensis*, CRISPR/Cas9, Sf-FGFR, Sf-SR-C

## Abstract

**Simple Summary:**

The CRISPR/Cas9 gene editing and biochemical analysis show that knocking out the *Sf-FGFR* or *Sf-SR-C* gene will not change the sensitivity of *Spodoptera frugiperda* to Vip3Aa.

**Abstract:**

Vip3Aa is a novel insecticidal protein secreted by *Bacillus thuringiensis* (*Bt*) during its vegetative growth stages. It has high insecticidal activity against lepidopteran pests such as *Spodoptera frugiperda*, and has no cross-resistance with Cry insecticidal proteins. As a new type of insecticide, it plays an important role in controlling agricultural pests. However, the insecticidal mechanism of the Vip3Aa toxin, especially its definite receptors, have not been fully revealed. In this study, the previously reported Vip3Aa receptor genes *Sf-FGFR* and *Sf-SR-C* were knocked out separately using the CRISPR/Cas9 system. Bioassay results showed that the sensitivity of these two knockout strains to Vip3Aa were not significantly changed compared to that of the normal strain. The current results are not consistent with the previously reports that Sf-SR-C and Sf-FGFR were the receptors of Vip3Aa in vitro. This suggests that the *Sf-SR-C* and *Sf-FGFR* genes we tested may not be critical in the mode of action of Vip3Aa in vivo in *Spodoptera frugiperda*.

## 1. Introduction

The fall armyworm, *Spodoptera frugiperda*, is one of the most devastating agricultural pests in the world and has more than 80 kinds of hosts, including maize, rice, sorghum, and cotton [1,2]. Though being native to tropical and subtropical America [3], *S. frugiperda* was successively discovered in Africa countries and Asia countries such as India, Thailand, and Myanmar, and caused serious economic losses [4,5,6,7]. At the end of 2018, *S. frugiperda* invaded Yunnan Province of China [8], and then spread rapidly to others provinces [9,10,11]. The rapid invasion of *S. frugiperda* seriously threatens global agricultural production security [12]. It is imperative to develop effective strategies for the management of *S. frugiperda*.

Since 1996, the use of transgenic crops expressing *Bacillus thuringiensis* (Bt) proteins has become an effective management method to control the devastating pest fall armyworm in many regions [13]. However, with the spreading cultivation of Bt crops, *S. frugiperda* has been reported to resist Cry proteins in some regions such as Puerto Rico, Florida, North Carolina, Brazil, and Argentina [14,15,16,17]. Vegetative insecticidal proteins (Vip) are produced by Bt and secreted during its vegetative stages. Although the activation mechanism of Vip3Aa have been elucidated, its insecticidal mechanisms is still unclear [18]. Moreover, the Vip3A protein is considered a novel insecticidal toxin as it does not share sequence or structural homology with known insecticidal crystal proteins [19]. As a novel toxin, the Vip3A protein shows higher insecticidal activity against a variety of lepidopteran pests such as *S. frugiperda* and have no cross-resistance to Cry proteins [20,21,22,23,24]. At present, Vip3A has been introduced into transgenic corn and cotton to expand the insecticidal spectrum and delay pest resistance to Cry toxins [25]. To data, *S. frugiperda* has not been observed to be resistant to Bt crops expressing the Vip3Aa protein in the field, but resistance alleles to Vip3A have been detected in the United States and Brazil [26,27]. Before pests develop widespread resistance to Vip3A, proactive implementation of resistance management strategies is particularly important to the sustainable use of Vip3A [28,29]. Therefore, analysis and confirmation of the unclear insecticidal mechanism and receptor of the Vip3A toxin will provide important information for resistance management strategies.

As for the receptor protein of Vip3A, analysis based on HPLC-MS/MS has shown that 70 proteins could bind to Vip3Aa in *S. frugiperda* (Sf9) cells, including scavenger receptor class C like protein (Sf-SR-C), fibroblast growth factor receptor-like protein (Sf-FGFR), and ribosomal S2 protein. Further analysis confirmed that the Sf-SR-C from Sf9 cells was a receptor protein of Vip3Aa through in vitro and in vivo biological experiments, and Sf-SR-C-mediated endocytosis of Vip3Aa was related to its insecticidal activity [30]. In addition, the study also found that Vip3Aa and Sf-FGFR can be internalized into Sf9 cells together, and downregulation of the *Sf-FGFR* gene can reduce the cytotoxicity of Vip3Aa. Therefore, Sf-FGFR from the membrane of Sf9 cells was identified as another receptor protein for Vip3Aa [31]. However, further verification is needed whether Sf-SR-C and Sf-FGFR are the receptor proteins of Vip3Aa in *S. frugiperda*.

In this study, we established knockout of the *Sf-SR-C* and *Sf-FGFR* genes of homozygous strains using the CRISPR/Cas9 gene-editing system, to investigate the role of the two genes in the insecticidal mechanism of Vip3Aa. Our data provide useful information for studying the insecticidal mechanism of Vip3Aa.

## 2. Materials and Methods

### 2.1. Insect Strains and Rearing

The DH19 susceptible strain of *S. frugiperda* in the study was initially collected from Dehong, Yunnan Province, China, in January 2019 [32]. The strain had been raised in laboratory conditions for more than two years without exposure to any toxins or pesticides, and is sensitive to both Bt toxins and chemical pesticides [33]. Two knockout strains of FGFR-KO and SRC-KO were established by using the CRISPR/Cas9 genome-editing tools to knock out the *Sf-SR-C* or *Sf-FGFR* genes of the DH19 strain. The larvae of all strains were fed with artificial diet based on wheat germ and soybean powder at 27 ± 2 °C, 75% ± 10% relative humidity, and a 14L:10D photoperiod. Adults were fed with a 10% sugar solution after emergence.

### 2.2. SgRNAs Design and Synthesis

The sgRNA of *Sf-SR-C*, *Sf-FGFR* gene was designed using sgRNA design software, and the off-target risk of sgRNA was evaluated using the whole genome of *S. frugiperda* as a reference sequence [34]. The N18NGG sequence with the highest score and no potential off-target sites was selected as the sgRNA target sequence. The *Sf-FGFR* target sequence (5′-TATGTGCAGCAGAAACATTGG-3′, the PAM sequence is underlined) at exon 1 of *Sf-FGFR*, and the *Sf-SR-C* target sequence (5′-TGTACTGTGATGGATCCAATTGG-3′, the PAM sequence is underlined) at exon 1 of *Sf-SR-C*. SgRNA was synthesized in vitro according to the instructions of the GeneArt™ Precision gRNA Synthesis Kit. Then the sgRNA was purified by the gRNA Clean Up Kit. Cas9 protein (GeneArt ™ Platinum ™ Cas9 Nuclease) was purchased from Thermo Fisher Scientific (Shanghai, China).

### 2.3. Collection and Injection of S. frugiperda Eggs

Eggs of *S. frugiperda* were collected and injected as described by Jin [32]. Briefly, fresh eggs were collected and soaked in a 1% sodium hypochlorite solution for 10 s before being washed with distilled water. The eggs were laid on a slide with double-sided tape. A 1 nL mixture of sgRNA (150 ng/μL) and Cas9 protein (50 ng/μL) was injected into a single egg using Nanoject III (Drummond, Broomall, USA). The injected eggs were placed in an incubator with a temperature of 25 °C and a relative humidity of 65% for incubation.

### 2.4. Genomic DNA Extraction and Mutagenesis Detection

Genomic DNA was extracted using the Multisource Genomic DNA Miniprep Kit. To determine the mutation types, specific primers of the *Sf-FGFR* gene (forward: 5’-GTGTGGCATGAAGCCCAGTA-3’, reverse: 5’-ATCCCCGATTCCCCAACAAC-3’) and *Sf-SR-C* gene (forward: 5’-TCGGGCTGCTCACAATTACA-3’, reverse: 5’-TGCAGAAAGGAACTCGGTGTC-3’) were designed. We then used PCR to amplify fragments flanking the CRISPR target sites. The PCR conditions were as reported by Jin [32]. PCR products were recovered, cloned, and sequenced by Sangon Biotech (Shanghai, China).

### 2.5. Bt Toxins and Bioassays

The Vip3Aa toxins used in this research were purchased from the Institute of Plant Protection, Chinese Academy of Agricultural Sciences (CAAS), Beijing, China. The sensitivity of two knockout strains and the DH19 strain to Vip3Aa toxin was determined by the diet overlay method. A gradient of concentrations of the Bt toxin solution was prepared with the Vip3Aa toxin dissolved in PBS solution with pH = 7 (3.125, 6.25, 12.5, 25, 50, 100, 200, and 400 ng/cm^2^). We added 900 μL of uncoagulated artificial diet to each well of the 24-well plate (surface area = 2 cm^2^). After the diet was cooled, we added 50 μL of the Bt toxin solution onto the surface of each well. A single newly hatched larvae was placed in each well after the toxin solution was dried at room temperature, and the mortality was recorded 7 days later. Apart from the larvae that died, those that weighed less than 5 mg at the end of the bioassay were also considered dead.

The median lethal concentration (LC_50_) and the corresponding 95% fiducial limits of each strain were calculated through probit analysis of the mortality data by DPS software. Two LC_50_ values were considered significantly different if their 95% fiducial limits did not overlap.

## 3. Results

### 3.1. Establishment of the Sf-FGFR Knockout Strain and Its Mutation Types

**Figure 1:**
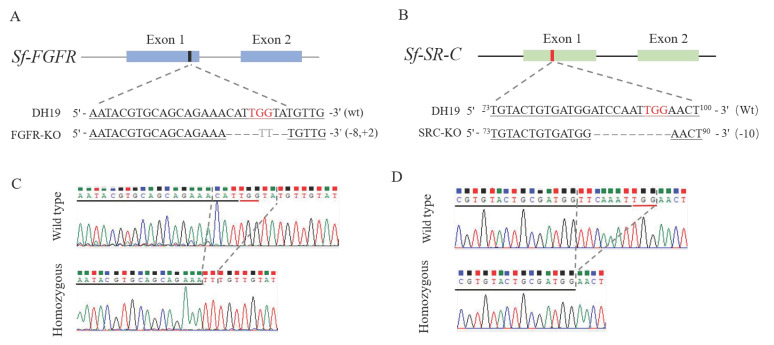
Sequence-specific mutation types of the *Sf-FGFR* and *Sf-SR-C* genes of *S. frugiperda* mediated by the CRISPR/Cas9 gene-editing system. (**A**,**B**) Schematic diagrams of sgRNA targeting sites and Sequence-specific mutation types of *Sf-FGFR* and *Sf-SR-C* genes. (**C**,**D**) Chromatograms of DNA sequences of selected homozygotes of *Sf-FGFR* and *Sf-SR-C*.

**Figure 2:**
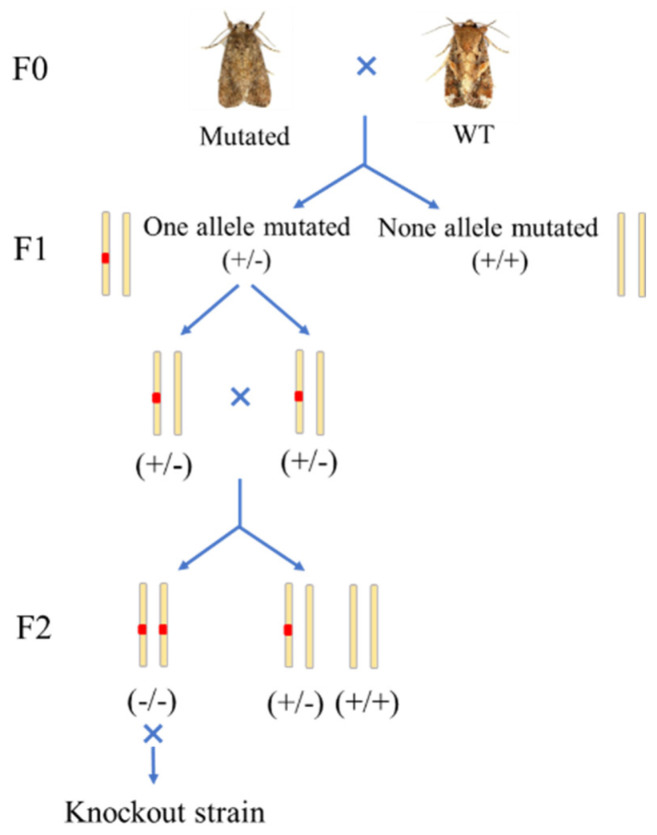
The procedures of FGFR−KO and SRC−KO establishment.

A total of 300 fresh eggs from *S. frugiperda* were sequentially injected with a mixture of Cas9 protein and the sgRNAs targeting exon 1 of the *Sf-FGFR* gene. About 15.3% (46/300) of the injected eggs hatched into larvae. The larvae were fed with artificial diet and 65.2% (30/46) of them successfully pupated. DNA was extracted from the exuvium of the larvae and PCR amplification assays revealed that 13.3% (4/30) of samples had deletion bands in the *Sf-FGFR* gene. These missing fragments were then sequenced to confirm the exact deletion genotype of the *Sf-FGFR* mutants. One mutant type with a deletion of 8-bp and an insertion of 2-bp at exon 1 of the *Sf-FGFR* gene was selected as the parent F0 generation to screen the homozygous line (Figure 1A), and the mutant individual was crossbred with the wild-type individual to produce the F1 progeny. F1 larvae were raised on artificial feed until pupation. Exuviae of 100 F1 generation larvae was used to extract DNA for mutation detection, and then 41 individuals containing the selected mutant gene were sibling-crossed to produce the F2 progeny. Homozygous mutation individuals were screened from 150 larval exuviae of F2 generation and then were sibling-crossed to establish the *Sf-FGFR* gene knockout strain FGFR-KO (Figure 2).

**Figure 3:**
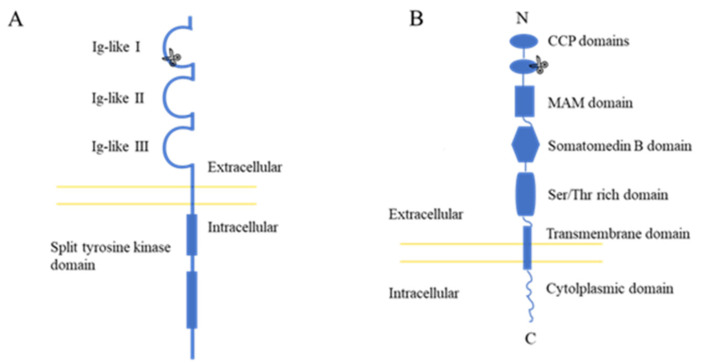
Basic structures of the FGFR (**A**) and SR-C (**B**). The figures were adapted from [34] and [35] respectively.

The FGFR (fibroblast growth factor receptor) consists mainly of three extracellular Ig-like domains and one intracellular tyrosine split kinase domain [35]. Our FGFR-KO strain deleted 8 bp and inserted 2 bp, resulting in two amino acids deletion in the Ig-like Ⅰ domain (Figure 3A). It should be mentioned that the mating and hatching rate of that the knockout strain FGFR-KO was decreased after rearing several generations.

### 3.2. Establishment of Sf-SR-C Knockout Strain and Its Mutation Types

Another sgRNA targeting exon 1 of the *Sf-SR-C* gene were mixed with Cas9 protein in a certain proportion, and then injected into 350 eggs of *S. frugiperda*. Among the injected eggs, 12.3% (43/350) hatched as larvae. Among 43 newly hatched larvae, 62.8% (27/43) developed into pupae. DNA was extracted from the exuvium of the larvae and PCR amplified analysis showed that 11.1% (3/27) of the samples had deletion bands in the *Sf-SR-C* gene. The exact mutation type of the *Sf-SR-C* gene was determined by sequencing. The mutant with a 10-bp deletion at exon 1 of the *Sf-SR-C* gene was selected as the parent F0 generation to screen the homozygous line (Figure 1B), and the knockout strain, named SRC-KO, was established as described in Section 2.1 (Figure 2).

The SR-C consists mainly of two extracellular N-terminal complementary control protein (CCP) domains, followed by an extracellular domain of the MAM family, a spacer, a Somatomedin B-like domain, a Ser/Thr-rich domain, a second spacer, a transmembrane domain, and finally a small intracellular cytolplasmic domain [36]. Our SRC-KO strain knocked out 10 bp in the second CCP domain (Figure 3B).

### 3.3. Susceptibility to Vip3Aa Toxins in FGFR-KO and SRC-KO

In order to determine whether the *Sf-FGFR* and *Sf-SR-C* genes are receptors for Vip3Aa in *S. frugiperda*, the sensitivity of the two knockout strains, FGFR-KO and SRC-KO, and the wild strain, DH19, to the Vip3Aa toxin was determined by diet overlay bioassays. The LC_50_ values of the knockout strains FGFR-KO and SRC-KO and the susceptible strain DH19 against the Vip3Aa toxin were 39.20 ng/cm^2^, 51.85 ng/cm^2^, and 36.64 ng/cm^2^, respectively, with no significant difference (Table 1). These results indicate that the sensitivity of *S. frugiperda* to the Vip3Aa toxin did not change significantly after the *Sf-FGFR* or *Sf-SR-C* genes were knocked out. Therefore, it was speculated that neither Sf-FGFR nor Sf-SR-C was the receptor for Vip3Aa to play an insecticidal role.

## 4. Discussion

Vip3A protein is a novel insecticidal protein produced by *Bacillus thuringiensis* at the vegetative growth stage, which does not have the same sequence homology and binding site as the Cry protein [19,37,38]. Compared with Cry1Ab and Cry1F, Vip3A showed higher insecticidal activity and high virulence to *S. frugiperda*. It was also reported that there was no cross-resistance between the Cry toxins and Vip3Aa [20,21,22,23]. Vip3Aa plays an important role in preventing and controlling agricultural pests and delaying the resistance of pests. Similar to the Cry protein, the Vip3A protein also needs to be activated by protease to exert its toxicity and bind to midgut cells, which, in turn, leads to cell apoptosis or pore formation [39]. However, so far, this complicated multi-step process has not been fully elucidated. In order to investigate the insecticidal mechanism of Vip3Aa in *S. frugiperda*, two genes of previously reported Vip3Aa receptors were knocked out from *S. frugiperda* by the CRISPR/Cas9 gene-editing system. Homozygous knockout strains of both genes were successfully obtained.

Fibroblast growth factor receptors (FGFRs) is an important member of the receptor tyrosine kinase (RTKs) family, which is mainly studied in mammalian cells [40,41]. It is composed of four members: FGFR1, FGFR2, FGFR3, and FGFR4. By combining with FGFs to perform biological functions, FGFRs have essential functions for maintaining normal cell growth, proliferation, and differentiation [42]. Relevant studies had found that the Sf-FGFR of *S. frugiperda* could be internalized into the Sf9 cells together with Vip3Aa. Sequence alignment revealed that SF-FGFR was most similar to FGFR1. Knockdown of the *Sf-FGFR* gene in Sf9 cells can reduce the cell’s mortality to Vip3Aa, indicating that Sf-FGFR was the receptor of Vip3Aa [31]. However, in our study, the sensitivity of the FGFR-KO strain to Vip3Aa was not significantly different compared with the susceptible strain DH19, which indicated that Sf-FGFR might not be the receptor of Vip3Aa in *S. frugiperda*. Compared with cultured cells, living worms have a more complex regulatory network [43]. Moreover, the Sf9 cells used in a previous study were derived from *S. frugiperda* ovary cells, whereas our in vivo results are testing the role of the two receptors in epithelial cells, which may have different functions. Another possibility is that FGFR is a gene family, and knockout of the *Sf-FGFR* gene in worms may cause other genes of the same family members to replace the function of the *Sf-FGFR* gene.

Another reported Vip3Aa receptor is the SR-C like protein Sf-SR-C from Sf9 cells and has been confirmed in *Spodoptera exigua* and *Drosophila* [30]. Scavenger receptors are a family of glycoproteins located on the surface of cell membranes and play an important role in host defense against pathogens [44]. According to the results of sequence alignment and protein domain characteristics, they were divided into ten subfamilies [45]. Scavenger receptor-C (SRC) has been identified only in a few invertebrates, and their role in the immune response is still poorly understood [46,47]. In mammals, SR proteins can trigger a series of signaling pathways through endocytosis [45,48]. Similarly, Sf-SR-C-mediated endocytosis was reported to be related to the toxicity of Vip3A to Sf9 cells [30]. In addition, the function of SR has been shown to be related to apoptosis of Sf9 cells, which agrees with the recent report that Vip3Aa can induce apoptosis of Sf9 cells [49,50,51,52,53,54]. However, our bioassay results showed that there was no significant change in susceptibility to Vip3Aa when the *Sf-SR-C* gene was knocked out from *S. frugiperda*, indicating that Sf-SR-C may not be involved in the toxicity of Vip3Aa in *S. frugiperda*. This result is contrary to previous studies that Sf-SR-C acts as a receptor for Bt vegetative insecticidal protein Vip3Aa, which may be because Vip3Aa receptors differ between species [55]. The fact that the receptors of insecticidal proteins differ between species has also been reported in Cry proteins. For example, cadherin was a toxic receptor for Cry1Ac in *Heliothis virescens*, *Helicoverpa armigera*, and *Platyedra gossypiella*, but not in *Spodoptera litura* and *Trichoplusia ni* [56,57,58,59,60,61].

To sum up, our results indicate that there was no significant change in sensitivity of *S. frugiperda* to Vip3Aa after knocking out the *Sf-FGFR* or *Sf-SR-C* gene, and thus that the mechanism of action of Vip3A is more complex. The reason for the inconsistencies between our in vivo and previously reported in vitro results may require further investigation.

## Figures and Tables

**Table 1:** Susceptibility of DH19, FGFR-KO, and SRC-KO strains to the Vip3Aa toxin.

Strain	N ^a^	LC_50_ (ng/cm^2^) ^b^	Slope ± SE ^c^	Toxicity Ratio ^d^
DH19	168	36.64 (22.52–59.61)	4.46 ± 0.65	1
FGFR-KO	168	39.20 (22.61–67.99)	4.44 ± 0.75	1.01
SRC-KO	168	51.85 (24.80–108.38)	4.80 ± 1.29	1.42

^a^ Number of larvae tested. ^b^ Concentration (ng/cm^2^) of Vip3Aa that kills 50% of larvae and its 95% fiducial limits. ^c^ Slope of the concentration-mortality line and its standard error. ^d^ Toxicity ratio = the LC_50_ of the knockout strain/the LC_50_ of the susceptible strain.

## Data Availability

The data presented in this study are available in article.

## References

[1] Montezano D.G., Specht A., Sosa-Gómez D.R., Roque-Specht V.F., Sousa-Silva J.C., Paula-Moraes S.V., Peterson J.A., Hunt T.E. (2018). Host Plants of *Spodoptera frugiperda* (Lepidoptera: Noctuidae) in the Americas. Afr. Entomol..

[2] Hardke J.T., Lorenz G.M., Leonard B.R. (2015). Fall Armyworm (Lepidoptera: Noctuidae) Ecology in Southeastern Cotton. Int. J. Pest Manag..

[3] Sparks A.N. (1979). A Review of the Biology of the Fall Armyworm. Fla. Entomol..

[4] Goergen G., Kumar P.L., Sankung S.B., Togola A., Tamò M. (2016). First Report of Outbreaks of the Fall Armyworm *Spodoptera frugiperda* (J E Smith) (Lepidoptera, Noctuidae), a New Alien Invasive Pest in West and Central Africa. PLoS ONE.

[5] Sisodiya D.B., Raghunandan B.L., Bhatt N.A., Verma H.S., Shewale C.P., Timbadiya B.G., Borad P.K. (2018). The fall armyworm, *Spodoptera frugiperda* (J.E. Smith) (Lepidoptera: Noctuidae); First report of new invasive pest in maize fields of Gujarat, India. J. Entomol. Zool. Stud..

[6] FAO (2018). First Detection of All Armyworm on the Border of Thailand.

[7] FAO (2019). First Detection Report of the Fall Armyworm Spodoptera frugiperda (Lepidoptera: Noctuidae) on Maize in MYANMAR.

[8] Guo J.F., He K.L., Wang Z.Y. (2019). Biological characteristics, trend of fall armyworm *Spodoptera frugiperda*, and the strategy for management of the pest. Chin. J. Appl. Entomol..

[9] Jiang Y.Y., Liu J., Xie M.C., Li Y.H., Yang J.J., Zhang M.L., Qiu K. (2019). Observation on law of diffusion damage of *Spodoptera frugiperda* in China in 2019. Plant Protect..

[10] Liao Y.L., Li C.Y., Huang S.H., Pan Z.P., Yang B., Chen J.R., Wang L.J., Liu W.L., Zhang Y.P. (2019). Survey on the prevalence and damage of *Spodoptera furgiperda* first invasive in Guangdong. J. Econ. Entomol..

[11] Wu Q., Jiang Y.Y., Wu K. (2019). Analysis of migration routes of the fall armyworm *Spodoptera frugiperda* (J. E. Smith) from Myanmar to China. Plant Protect..

[12] Stokstad E. (2017). New crop pest takes Africa at lightning speed. Science.

[13] ISAAA (2017). Global Status of Commercialized Biotech/GM Crops in 2017: Biotech Crop Adoption Surges as Economic Benefits Accumulate in 22 Years.

[14] Storer N.P., Babcock J.M., Schlenz M., Meade T., Thompson G.D., Bing J.W., Huckaba R.M. (2010). Discovery and characterization of field resistance to Bt maize: *Spodoptera frugiperda* (Lepidoptera: Noctuidae) in Puerto Rico. J. Econ. Entomol..

[15] Huang F., Qureshi J.A., Head G.P., Price P.A., Levy R., Yang F., Niu Y. (2016). Frequency of *Bacillus thuringiensis* Cry1A.105 resistance alleles in field populations of the fall armyworm, *Spodoptera frugiperda*, in Louisiana and Florida. Crop Prot..

[16] Monnerat R., Martins E., Macedo C., Queiroz P., Praça L., Soares C.M., Moreira H., Grisi I., Silva J., Soberon M. (2015). Evidence of field-evolved resistance of *Spodoptera frugiperda* to Bt corn expressing Cry1F in Brazil that is still sensitive to modified Bt toxins. PLoS ONE.

[17] Chandrasena D.I., Signorini A.M., Abratti G., Storer N.P., Olaciregui M.L., Alves A.P., Pilcher C.D. (2018). Characterization of field-evolved resistance to *Bacillus thuringiensis*-derived Cry1F δ-endotoxin in *Spodoptera frugiperda* populations from Argentina. Pest Manag. Sci..

[18] Núñez-Ramírez R., Huesa J., Bel Y., Ferré J., Casino P., Arias-Palomo E. (2020). Molecular architecture and activation of the insecticidal protein Vip3Aa from *Bacillus thuringiensis*. Nat. Commun..

[19] Estruch J.J., Warren G.W., Mullins M.A., Nye G.J., Craig J.A., Koziel M.G. (1996). Vip3A, a novel *Bacillus thuringiensis* vegetative insecticidal protein with a wide spectrum of activities against lepidopteran insects. Proc. Natl. Acad. Sci. USA.

[20] Sena J.A., Hernández-Rodríguez C.S., Ferré J. (2018). Interaction of *Bacillus thuringiensis* Cry1 and Vip3A proteins with *Spodoptera frugiperda* midgut binding sites. Appl. Environ. Microbiol..

[21] Yang F., Huang F.N., Qureshi J.A., Leonard B.R., Niu Y., Zhang L.P., Wangila D.S. (2013). Susceptibility of Louisiana and Florida populations of *Spodoptera frugiperda* (Lepidoptera:Noctuidae) to transgenic Agrisure (R) Viptera (TM) 3111 corn. Crop Prot..

[22] Yang F., Kerns D.L., Head G., Brown S., Huang F.N. (2017). Susceptibility of Cry1F-maize resistant, heterozygous, and susceptible *Spodoptera frugiperda* to Bt proteins used in the transgenic cotton. Crop Prot..

[23] Yang F., Kerns D.L., Head G.P., Price P., Huang F. (2017). Cross-resistance to purified Bt proteins, Bt corn and Bt cotton in a Cry2Ab2-corn resistant strain of *Spodoptera frugiperda*. Pest Manag. Sci..

[24] Gomis-Cebolla J., Wang Y., Quan Y., He K., Walsh T., James B., Downes S., Kain W., Wang P., Leonard K. (2018). Analysis of cross-resistance to Vip3 proteins in eight insect colonies, from four insect species, selected for resistance to *Bacillus thuringiensis* insecticidal proteins. J. Invertebr. Pathol..

[25] Chakroun M., Banyuls N., Bel Y., Escriche B., Ferré J. (2016). Bacterial Vegetative Insecticidal Proteins (Vip) from Entomopathogenic Bacteria. Microbiol. Mol. Biol. Rev..

[26] Bernardi O., Bernardi D., Ribeiro R.S., Okuma D.M., Salmeron E., Fatoretto J., Medeiros F.C., Burd T., Omoto C. (2015). Frequency of resistance to Vip3Aa20 toxin from *Bacillus thuringiensis* in *Spodoptera frugiperda* (Lepidoptera: Noctuidae) populations in Brazil. Crop Prot..

[27] Yang F., Williams J., Porter P., Huang F., Kerns D.L. (2019). F2 screen for resistance to *Bacillus thuringiensis* Vip3Aa51 protein in field populations of *Spodoptera frugiperda* (Lepidoptera: Noctuidae) from Texas, USA. Crop Prot..

[28] Fitt G.P. (2000). An Australian approach to IPM in cotton: Integrating new technologies to minimise insecticide dependence. Crop Prot..

[29] Tabashnik B.E., Mota-Sanchez D., Whalon M.E., Hollingworth R.M., Carrière Y. (2014). Defning terms for proactive management of resistance to Bt crops and pesticides. J. Econ. Entomol..

[30] Jiang K., Hou X.Y., Tan T.T., Cao Z.L., Mei S.Q., Yan B., Chang J., Han L., Zhao D., Cai J. (2018). Scavenger receptor-C acts as a receptor for *Bacillus thuringiensis* vegetative insecticidal protein Vip3Aa and mediates the internalization of Vip3Aa via endocytosis. PLoS Pathog..

[31] Jiang K., Hou X., Han L., Tan T., Cao Z., Cai J. (2018). Fibroblast Growth Factor Receptor, a Novel Receptor for Vegetative Insecticidal Protein Vip3Aa. Toxins.

[32] Jin M., Tao J., Li Q., Cheng Y., Sun X., Wu K., Xiao Y. (2019). Genome editing of the SfABCC2 gene confers resistance to Cry1F toxin from *Bacillus thuringiensis* in *Spodoptera frugiperda*. J. Integr. Agric..

[33] Li G.P., Sun X.X., Jiang Y.Y., Wu K.M., Feng H.Q. (2019). Susceptibility evaluation of invaded *Spodoptera frugiperda* population in Yunan province to five Bt proteins. Plant Protect..

[34] Xie S., Shen B., Zhang C., Huang X., Zhang Y. (2014). sgRNAcas9: A software package for designing CRISPR sgRNA and evaluating potential off-target cleavage sites. PLoS ONE.

[35] Wesche J., Haglund K., Haugsten E.M. (2011). Fibroblast growth factors and their receptors in cancer. Biochem. J..

[36] Pearson A.M. (1996). Scavenger receptors in innate immunity. Curr. Opin. Immunol..

[37] Lee M.K., Walters F.S., Hart H., Palekar N., Chen J.S. (2003). The mode of action of the *Bacillus thuringiensis* vegetative insecticidal protein Vip3A differs from that of Cry1Ab delta-endotoxin. Appl. Environ. Microbiol..

[38] Lee M.K., Miles P., Chen J.S. (2006). Brush border membrane binding properties of *Bacillus thuringiensis* Vip3A toxin to *Heliothis virescens* and *Helicoverpa zea* midguts. Biochem. Biophys. Res. Commun..

[39] Syed T., Askari M., Meng Z., Li Y., Abid M.A., Wei Y., Guo S., Liang C., Zhang R. (2020). Current Insights on Vegetative Insecticidal Proteins (Vip) as Next Generation Pest Killers. Toxins.

[40] Dai S., Zhou Z., Chen Z., Xu G., Chen Y. (2019). Fibroblast Growth Factor Receptors (FGFRs): Structures and Small Molecule Inhibitors. Cells.

[41] Katoh M. (2016). Therapeutics Targeting FGF Signaling Network in Human Diseases. Trends Pharmacol. Sci..

[42] Turner N., Grose R. (2010). Fibroblast growth factor signalling: From development to cancer. Nat. Rev. Cancer.

[43] Bel Y., Jakubowska A.K., Costa J., Herrero S., Escriche B. (2013). Comprehensive analysis of gene expression profiles of the beet armyworm *Spodoptera exigua* larvae challenged with *Bacillus thuringiensis* Vip3Aa toxin. PLoS ONE.

[44] PrabhuDas M.R., Baldwin C.L., Bollyky P.L., Bowdish D.M.E., Drickamer K., Febbraio M., Herz J., Kobzik L., Krieger M., Loike J. (2017). A Consensus Definitive Classification of Scavenger Receptors and Their Roles in Health and Disease. J. Immunol..

[45] Canton J., Neculai D., Grinstein S. (2013). Scavenger receptors in homeostasis and immunity. Nat. Rev. Immunol..

[46] Pearson A., Lux A., Krieger M. (1995). Expression cloning of dSR-CI, a class C macrophage-specific scavenger receptor from Drosophila melanogaster. Proc. Natl. Acad. Sci. USA.

[47] Yang M.C., Shi X.Z., Yang H.T., Sun J.J., Xu L., Wang X.W., Zhao X.F., Wang J.X. (2016). Scavenger Receptor C Mediates Phagocytosis of White Spot Syndrome Virus and Restricts Virus Proliferation in Shrimp. PLoS Pathog..

[48] Yu X., Guo C., Fisher P.B., Subjeck J.R., Wang X.Y. (2015). Scavenger Receptors: Emerging Roles in Cancer Biology and Immunology. Adv. Cancer Res..

[49] Zhu X.D., Zhuang Y., Ben J.J., Qian L.L., Huang H.P., Bai H., Sha J.H., He Z.G., Chen Q. (2011). Caveolae-dependent endocytosis is required for class A macrophage scavenger receptor-mediated apoptosis in macrophages. J. Biol. Chem..

[50] Li K., Yang M., Yuen P.M., Chik K.W., Li C.K., Shing M.M., Lam H.K., Fok T.F. (2003). Thrombospondin-1 induces apoptosis in primary leukemia and cell lines mediated by CD36 and Caspase-3. Int. J. Mol. Med..

[51] Murphy J.E., Tacon D., Tedbury P.R., Hadden J.M., Knowling S., Sawamura T., Peckham M., Phillips S.E., Walker J.H., Ponnambalam S. (2006). LOX-1 scavenger receptor mediates calcium-dependent recognition of phosphatidylserine and apoptotic cells. Biochem. J..

[52] Oka K., Sawamura T., Kikuta K., Itokawa S., Kume N., Kita T., Masaki T. (1998). Lectin-like oxidized low-density lipoprotein receptor 1 mediates phagocytosis of aged/apoptotic cells in endothelial cells. Proc. Natl. Acad. Sci. USA.

[53] Jiang K., Mei S.Q., Wang T.T., Pan J.H., Chen Y.H., Cai J. (2016). Vip3Aa induces apoptosis in cultured *Spodoptera frugiperda* (*Sf9*) cells. Toxicon.

[54] Hou X., Han L., An B., Zhang Y., Cao Z., Zhan Y., Cai X., Yan B., Cai J. (2020). Mitochondria and Lysosomes Participate in Vip3Aa-Induced *Spodoptera frugiperda Sf9* Cell Apoptosis. Toxins.

[55] Jurat-Fuentes J.L., Heckel D.G., Ferré J. (2021). Mechanisms of Resistance to Insecticidal Proteins from *Bacillus thuringiensis*. Annu. Rev. Entomol..

[56] Gahan L.J., Gould F., Heckel D.G. (2001). Identification of a gene associated with Bt resistance in *Heliothis virescens*. Science.

[57] Wang J., Zhang H., Wang H., Zhao S., Zuo Y., Yang Y., Wu Y. (2016). Functional validation of cadherin as a receptor of Bt toxin Cry1Ac in *Helicoverpa armigera* utilizing the CRISPR/Cas9 system. Insect Biochem. Mol. Biol..

[58] Wang G., Wu K., Liang G., Guo Y. (2005). Gene cloning and expression of cadherin in midgut of *Helicoverpa armigera* and its Cry1A binding region. Sci. China C Life Sci..

[59] Qiu L., Hou L., Zhang B., Liu L., Li B., Deng P., Ma W., Wang X., Fabrick J.A., Chen L. (2015). Cadherin is involved in the action of *Bacillus thuringiensis* toxins Cry1Ac and Cry2Aa in the beet armyworm, *Spodoptera exigua*. J. Invertebr. Pathol..

[60] Ma Y., Zhang J., Xiao Y., Yang Y., Liu C., Peng R., Yang Y., Bravo A., Soberón M., Liu K. (2019). The cadherin Cry1Ac binding-region is necessary for the cooperative effect with ABCC2 transporter enhancing insecticidal activity of *Bacillus thuringiensis* Cry1Ac toxin. Toxins.

[61] Wang S., Kain W., Wang P. (2018). *Bacillus thuringiensis* Cry1A toxins exert toxicity by multiple pathways in insects. Insect Biochem. Mol. Biol..

